# Ruptured Anterior Tibial Artery Pseudoaneurysm following Ilizarov External Fixator: A Case Report

**DOI:** 10.5704/MOJ.2311.015

**Published:** 2023-11

**Authors:** SA Zakaria, NA Yacob

**Affiliations:** 1 Department of Orthopaedics, Traumatology and Rehabilitation, International Islamic University of Malaysia (IIUM), Kuantan, Malaysia; 2 Department of Orthopaedics, Hospital Sultanah Nur Zahirah, Kuala Terengganu, Malaysia

**Keywords:** pseudoaneurysm, Ilizarov procedure, vascular injury

## Abstract

Genuine infrapopliteal aneurysms are quite rare, in contrast to pseudoaneurysms. The aetiology of pseudoaneurysms related to external fixation is attributed to various theories, including direct vascular damage due to misplaced pins or wires, overshooting or misguidance during osteotomy, distraction at the corticotomy site during an Ilizarov procedure, and continuous abrasion of the vessel caused by a wire inserted in close proximity to an artery. Arteriography proves valuable in documenting lesions and assessing deeper pseudoaneurysms, particularly when contemplating reconstruction; it plays a crucial role in guiding management decisions. For significant tears and symptomatic aneurysms, resection and reconstruction are the gold standard treatment.

## Introduction

Genuine infrapopliteal aneurysms are indeed quite rare, whereas false aneurysms or pseudoaneurysms are more commonly encountered^1^. Aneurysms affecting large blood vessels such as the aorta, carotid, femoral, and popliteal arteries are more prevalent and are primarily attributed to generalised atherosclerotic disease^2^. In contradistinction to true aneurysms, pseudoaneurysms lack all three layers of the vessel wall. They arise in association with penetrating injuries that lead to transmural laceration of the arterial wall^3^. External fixators are recognised for their potential to cause complications related to the insertion of pins or guidewires, and one of these complications is the development of pseudoaneurysms^4^.

We present a case involving a ruptured pseudoaneurysm of the anterior tibial artery subsequent to an Ilizarov procedure. The clinical manifestations, diagnostic evaluations, and treatment strategies will be elaborated upon.

## Case Report

**Fig 1:**
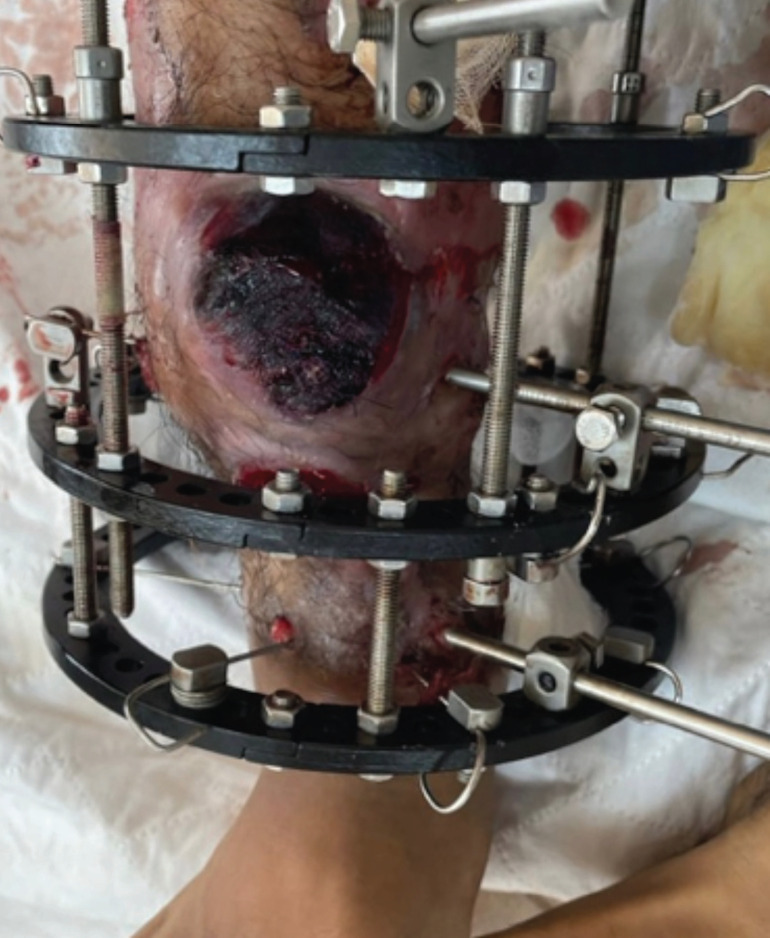
Pulsatile swelling over distal 3rd right anterior shin.

We present a case involving an 18-year-old male patient who was admitted to the orthopaedic ward due to bleeding from the anterior right distal shin. His medical history includes a traumatic incident 10 months before admission, resulting in an open comminuted fracture of the distal third of the right tibia and fibula, accompanied by a 5cm bone loss (classified as Gustillo Anderson 3B). Initially, he underwent bone debridement, and external fixation was applied to the anteromedial side of the tibia. A cement block was utilised to bridge the bone gap. Subsequently, five months later, he underwent Ilizarov external fixation and bone transport for the right tibia. The immediate post-operative period was uneventful until his current presentation, which occurred five months after the procedure. At this point, he sought medical attention at a district hospital due to persistent bleeding from the Ilizarov pin site, which had been ongoing for three weeks. Three days after his visit, he experienced a significant bleeding episode from the right leg after a routine dressing change, leading to his transfer to our medical centre (Fig. 1). Upon admission, urgent computed tomography (CT) angiography of the right lower limb was conducted; however, the study's quality was compromised by artifacts from the Ilizarov external fixator, rendering it unable to identify the source of bleeding effectively. Initially, upon admission, the bleeding was managed and controlled with a compression bandage. Nevertheless, over time, the dressing became frequently soaked. During a dressing change in the ward, a substantial spurting of blood from the distal third of the right anterior shin was observed, accompanied by an expanding pulsatile hematoma. The patient also exhibited symptoms of anaemia, such as lethargy and palpitations.

**Fig 2:**
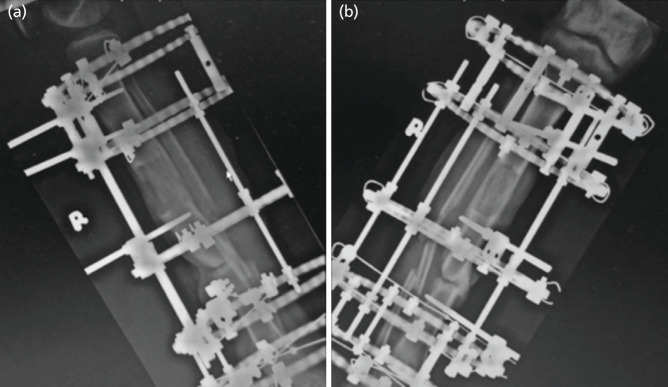
Radiograph of right tibia fibula, (a) lateral view and (b) anterior posterior view.

Upon examination, the patient presented with pallor and tachycardia. Blood pressure levels remained borderline normotensive, fluctuating between systolic values of 85 to 100 mmHg and diastolic readings ranging from 54 to 65 mmHg. On the distal third of the right anterior shin, a pulsatile swelling measuring 5x3cm was observed, accompanied by another swelling measuring 2x3cm distally. Pulsatile bleeding was observed and temporarily halted through compression. The area was temporarily sutured using *Vicryl 2/0*. Palpation of the right dorsalis pedis artery and posterior tibialis artery revealed good volume and comparability to the contralateral side. All toes displayed pink, and capillary refill time was less than two seconds. An admission radiograph of the right tibia and fibula showed partial union at the docking site, along with progressing osteogenesis at the consolidation site (Fig. 2). Prompt initiation of blood transfusion was undertaken. A COVID-19 rapid antigen test was administered as the patient was scheduled for an urgent operation, revealing a positive result. Consequently, he was isolated, and the Ilizarov external fixator was removed at the bedside in preparation for a repeat CT angiography of the right lower limb.

**Fig 3:**
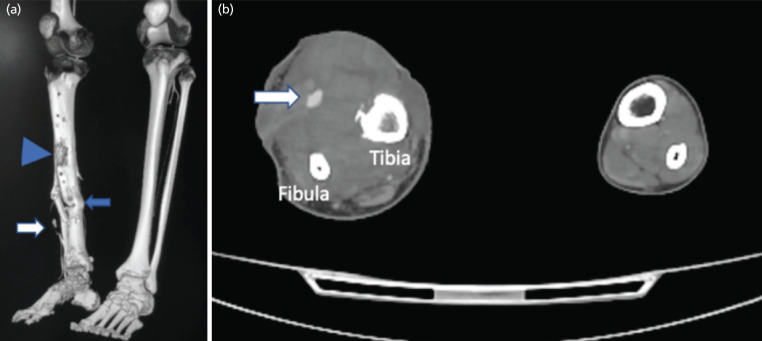
(a) CT angiography right lower limb. The white arrow showing the location of aneurysm. The blue arrow is the docking site. The arrow head is the new regenerated bone. (b) Image showing the aneurysm (white arrow) has been pushed more anteriorly by the hematoma.

The subsequent CT angiography of the right lower limb revealed a saccular aneurysm of the right anterior tibial artery, situated approximately 8.5cm above the tibiotalar joint line. The aneurysm pointed medially and measured 1.0cm x 0.7cm x 1.6cm (AP x W x CC) in dimensions, with an adjacent hematoma evident (indicated by the white arrow, Fig. 3). This aneurysm was positioned just below the docking site and had been displaced more anteriorly due to hematoma accumulation. The dorsalis pedis artery and popliteal artery displayed satisfactory opacification. Subsequently, an emergency procedure was carried out on the following day under COVID-19 protocol. The operation encompassed wound exploration, ligation, and resection of the right anterior tibial artery aneurysm. Intra-operatively, multiple pinpoint bleeding sites were identified on the aneurysm. Post-aneurysm resection, a 12cm segment of the anterior tibial artery exhibited a defect. This segment was subsequently excised, while sufficient collateral circulation was noted in the surrounding area. The intra-operative estimated blood loss totalled 2.5 litres, and the patient underwent 7 units of packed cell transfusion, including 4 units pre-operatively. The patient's condition remained stable, and on the third day post-operation, he was transferred to the general ward.

## Discussion

A traumatic pseudoaneurysm is formed by the reorganisation of a hematoma resulting from a partial artery disruption, leading to the accumulation of blood within surrounding tissues. The presence of an intact segment of the arterial wall prevents constriction of the vessel and thus mitigates uncontrolled extravasation, while turbulent blood flow persists in the central region^3^.

The characteristic presentation often involves an enlarging, pulsating hematoma accompanied by a systolic bruit and thrill, although these features may also be lacking^4^. As reported, distal pulsations are commonly detected, potentially attributable to the pulse pressure traversing an intimal flap, a soft and recent clot, or through collateral vessels. Furthermore, owing to the incomplete disruption of the vessel wall, a certain degree of blood flow persists beyond the aneurysmal site, contributing to the presence of an occasionally faint but perceptible distal pulse^3^.

The initial theory proposed to explain the aetiology of pseudoaneurysms associated with external fixation was based on the concept of direct vascular injury resulting from improperly positioned pins or wires^5^. Clinical signs may manifest within a few days to weeks. The incidence of neurovascular injury associated with the insertion of half-pins for external fixation has been reported to be as low as 0.6% in a study encompassing over 4,000 trauma cases treated with external fixation. This incidence has been maintained at that level due to rigorous adherence to the principles of external fixation outlined in the study by Behrens and Searls^5^.

Additionally, vascular injury could have stemmed from an excessive or improperly directed use of an osteotome, as well as from distraction at the corticotomy site following an Ilizarov procedure^3^. Initially, blood clots might have occluded the fracture, but subsequent distraction at the corticotomy site could have led to the separation of the fracture, allowing for subsequent leakage and the eventual formation of a pseudoaneurysm^3^.

When a wire is implanted in close proximity to an artery, friction against the vessel occurs over time due to continuous arterial pulsations within a relatively static environment^3^. Jakim *et al.*^4^ suggest that the longitudinal micro-movement of a transfixation wire against the artery's wall could exacerbate ongoing attrition, leading to the delayed emergence of pseudoaneurysms, as observed in this case. The utilisation of thin wires renders them particularly susceptible to iatrogenic damage. Therefore, it is recommended to employ half pins instead of transfixing wires in this anatomical region^3^.

In this instance, we have considered two potential scenarios that could have caused the initial injury to the anterior tibial artery, illustrated in (Fig. 1 and Fig. 2). The hydroxyapatite (HA) coated pin was introduced from the anteromedial aspect toward the posterolateral side, just below the docking site. Positioned below was a tensioned wire crossing transversely, with a lateral and medial aspect, with the medial wire inserted just anterior to the tibia's medial edge, extending toward the anterolateral corner of the tibia. Typically, these two wires are situated anterior to the midline of the tibia in the sagittal plane. Nevertheless, multiple attempts at inserting the wire may elevate the risk of vessel injury, particularly concerning the medial wire. Another potential scenario involves the HA coated pin inserted from the medial aspect. The primary cause of vessel injury usually stems from excessive drill over-drilling. Notably, intra-operatively, there were no indications of arterial injury, and the post-operative course proceeded without any noteworthy incidents.

After experiencing trauma, pseudoaneurysms can sometimes remain undetected, especially during the interval preceding rupture. In cases where the presentation of vascular damage symptoms is delayed, vigilant and continuous monitoring of the site of injury becomes essential. As seen in our patient, symptoms only became apparent five months after the bone transport surgery. Arteriography plays a pivotal role in assessing pseudoaneurysms located deeper within the tissues and in documenting any associated lesions. This diagnostic approach gains significance when contemplating the need for reconstructive interventions and may play a crucial role in determining whether to proceed with ligation or repair of the affected artery^3^.

The approach to treating arterial pseudoaneurysms is determined by their location, appearance, and whether there is an infection present^3^. In cases where the diameter is around 10mm, and they involve minor arteries, close observation might be sufficient, especially if they are asymptomatic. However, intervention is typically required for larger, symptomatic pseudoaneurysms or those affecting major arteries where blockage could lead to significant ischemic consequences. These cases might necessitate surgical intervention or could potentially be managed by an interventional radiologist to induce pseudoaneurysm thrombosis. Neglecting treatment poses a significant risk of enlargement and eventual rupture, potentially leading to fatal outcomes. For substantial tears, the gold standard of treatment is resection and reconstruction. Amputation is considered a last-resort option, usually in cases of severe infection, life-threatening bleeding, or as a worst-case scenario^3^.

In our specific case, we opted for ligation due to ongoing bleeding from multiple points of the aneurysm. Unfortunately, our medical facility lacks the capability for embolisation, and the nearest available centre for this procedure is three hours away. Complicating matters, the patient has tested positive for COVID-19, which has introduced a delay in treatment. Ultimately, given the intact posterior tibial artery, the decision to ligate the anterior tibial artery was considered safe, as it would not compromise blood supply to the affected limb.

An additional challenge we must address in this case involves the complexities of treating a patient who has tested positive for COVID-19. The principles of prioritising life over limbs must guide our decisions. A swift and comprehensive assessment becomes paramount in determining the optimal and most definitive treatment strategy for the patient, all while ensuring the safety of healthcare providers is not compromised. Balancing the medical needs of the patient with the risk of COVID-19 transmission adds a layer of complexity to the decision-making process
